# The effects on pain and disability of traditional Chinese non-pharmacological therapy for knee osteoarthritis: A protocol for systematic review and meta-analysis

**DOI:** 10.1097/MD.0000000000027005

**Published:** 2021-08-27

**Authors:** Wei Chang, Weina Guo, Rongjun Wang, Xingxi Lin, Sifei Sun, Yibin Shi

**Affiliations:** Community Healthcare Center of Jiangqiao Town, Shanghai, China.

**Keywords:** disability, knee osteoarthritis, pain, traditional Chinese non-pharmacological therapy

## Abstract

**Background:**

Knee osteoarthritis (KOA) is a common chronic joint disease with serious health economic burden. More and more randomized controlled trials have indicated that traditional Chinese non-pharmacological therapy, including acupuncture, Tai Chi, Tuina, etc can significantly improve pain and physical function of patients with KOA. However, the effects of traditional Chinese non-pharmacological therapy for KOA remain controversial. Most previous systematic reviews did not focus on the effects of traditional Chinese non-pharmacological therapy for KOA as a whole. In Chinese community hospital, however, acupuncture, Tuina, and Tai Chi are usually in the management of KOA as whole-body treatment.

**Methods:**

The electronic databases (PubMed, Embase, MEDLINE, Cochrane Central Register of Controlled Trials, Web of Science, China Knowledge Resource Integrated Database, and Wanfang Data) will be searched. The search will include all documents from their inception to December 2021. Two reviewers independently extracted the data and assessed the risk of bias by the Cochrane Risk of Bias Tool for randomized controlled trials. The meta-analysis will be conducted with a random or fixed effect model to calculate the standardized mean difference and 95% confidence intervals based on different heterogeneity using the Review Manager Version 5.3 software. The heterogeneity will be examined by Higgins I2 statistic. The subgroup analysis will be conducted based on different types of traditional Chinese non-pharmacological therapy and different outcomes. Quality of evidence will be assessed using the Grades of Recommendation, Assessment, Development and Evaluation.

**Results:**

The current systematic review and meta-analysis will be conducted to investigate the effects of traditional Chinese non-pharmacological therapy in the management of KOA. The main outcomes will include pain and disability. The secondary outcomes will include quality of life and adverse events.

**Conclusion:**

To provide evidence for evidence-based medicine and clinical researchers to choose more effective traditional Chinese non-pharmacological therapy for KOA.

**INPLASY registration number:**

INPLASY202170098.

**Strengths and Limitations of this Study**

- The current systematic review and meta-analysis will investigate the effects of traditional Chinese non-pharmacological therapy in the management of KOA, focusing on pain and disability of patients with KOA.
- The systematic review will only include the evidence from randomized controlled trials of traditional Chinese non-pharmacological therapy including acupuncture, Tuina, Tai Chi, Qigong, Baduanjin, Yijinjing, Liuzijue, and Wuqinxi for KOA.

## 1 Introduction

Knee osteoarthritis (KOA) is a common chronic joint disease characterized by joint pain and functional limitation worldwide. The prevalence of KOA is expected to increase due to increased aging and rates of obesity of the global population.^[1]^ In the United States about 14 million people suffered from pain and disability due to KOA.^[2]^ In Korea, the prevalence of radiographic KOA is 35.1% (24.4% in men, 44.3% in women) in adults over 50 years, and the prevalence is up to 78.7% in women over 80 years.^[3]^ In China, the prevalence rate of KOA is 8.1%, and the prevalence rate is increasing rapidly among people older than 45 years.^[4,5]^ According to the Global Burden of Diseases study, the burden of osteoarthritis, especially KOA, is increasing, which will impose new challenges on the health systems, along with mental disorders and diabetes.^[6]^

About the complementary and alternative therapy of KOA, the Osteoarthritis Research Society International (OARSI) and European Society for Clinical and Economic Aspects of Osteoporosis, Osteoarthritis and Musculoskeletal Diseases (ESCEO) both recommend that patient education, exercise, and weight loss (if a patient is overweight) should form the core treatment approach.^[7]^ Non-pharmacological intervention may be an important option for patients with KOA. In China, traditional Chinese non-pharmacological therapies, including acupuncture, Tuina, Tai Chi, etc are mostly used in the management of KOA. Acupuncture showed beneficial effects in reducing pain due to KOA.^[8]^ Tuina, as a manual therapy, could improve functional limitation of knee joint.^[9,10]^ The OARSI guidelines also recommend Tai Chi for the first-line treatment of KOA.^[11]^ Traditional Chinese non-pharmacological therapy may be beneficial for limb motor function, balance function, and daily life activity.^[12]^

Currently, the meta-analysis have reported that Tai Chi may be effective for KOA.^[13]^ More and more randomized controlled trials (RCT) have indicated that acupuncture can significantly alleviate pain, relieve stiffness, and improve physical function of patients with KOA.^[14,15]^ However, the effects of traditional Chinese non-pharmacological therapy for KOA remains controversial. Several trials could not suggest the effects of traditional Chinese non-pharmacological therapy for KOA due to the small sample size, short duration time, etc.^[16]^ Furthermore, previous systematic review and meta-analysis did not focus on the effects of traditional Chinese non-pharmacological therapy for KOA as a whole. In Chinese community hospital, however, acupuncture, Tuina, and Tai Chi are usually in the management of KOA as whole-body treatment.^[17,18]^

Therefore, the current systematic review and meta-analysis will be to investigate the effects of traditional Chinese non-pharmacological therapy in the management of KOA. The primary outcomes will be focused on pain and disability of patients with KOA.

## 2 Methods

### 2.1 Study registration

This protocol of systematic review and meta-analysis has been drafted according to the preferred reporting items for systematic reviews and meta-analysis, and was registered on the international platform of registered systematic review and meta-analysis protocols (the registration number: INPLASY202170098) on July 31, 2021.

The systematic review and meta-analysis will not require ethical approval because there are no patient recruitment and personal information collection.

### 2.2 Eligibility criteria

#### 2.2.1 Type of studies

In this meta-analysis, only RCT of traditional Chinese non-pharmacological therapy in the management of KOA will be included. Language will be restricted to English and Chinese.

#### 2.2.2 Types of participants

Participants with diagnosis of KOA by the American College of Rheumatology, the American Rheumatism Association, radio-graphic evidence or physician-confirmed diagnosis.^[19,20]^ There were no limitations on age, sex, or nationality of patients with KOA.

#### 2.2.3 Intervention and comparisons

In treatment group, patients with KOA were treated by traditional Chinese non-pharmacological therapy including acupuncture, moxibustion, Tuina, Tai Chi, Baduanjin, Wuqinxi, Liuzijue, and Yijinjing, which was used alone or in combination (such as Tuina plus acupuncture). In control group, the patients were treated by medicine, observation, education, sham exercise, sham acupuncture, and so on.

#### 2.2.4 Outcomes

Primary outcomes are pain (assessing by visual analogue scale score or any other valuable scales) and disability (assessing by the Western Ontario and McMaster university orthopedic index, Knee Injury and Osteoarthritis Outcome Score, or any other valuable scales). The secondary outcomes will include quality of life (assessing by 36-Item Short Form Survey or any other valuable scales) and adverse events.

### 2.3 Search strategy

Two reviewers will independently perform a systematic search of PubMed, Embase, MEDLINE, Cochrane Central Register of Controlled Trials, Web of Science, China Knowledge Resource Integrated Database, and Wanfang Data from their inception to December 2021. The search terms include “knee osteoarthritis”; “acupuncture” or “Tuina” or “Chinese massage” or “moxibustion” or “Taiji” or “Tai Chi” or “Baduanjin” or “Wuqinxi” or “Yijinjing” or “Liuzijue”; “pain” or “disability” or “functional limitation.” The search strategy details for PubMed are presented in Table 1. The similar terms will be translated into Chinese for Chinese databases. The language will be limited to English and Chinese.

**Table 1:** Search strategy for PubMed.

Search query	
#1	Search “ knee osteoarthritis ”[tiab]
#2	Search “ Taiji ”[tiab] OR “ Tai Chi ”[tiab] OR “ acupuncture ”[tiab] OR “ moxibustion ”[tiab] OR “ Baduanjin ”[tiab] OR “ Wuqinxi ”[tiab] OR “ Yijinjing ”[tiab] OR “ Liuzijue ”[tiab] OR “ Tuina ”[tiab] OR “ Chinese massage ”[tiab]
#3	Search “ pain ”[tiab] OR “ disability ”[tiab] OR “ functional limitation ”[tiab]
#4	Search “randomized clinical trial”[Article type]
#5	Search #1 AND #2 AND #3 AND #4

### 2.4 Study selection

**Figure 1:**
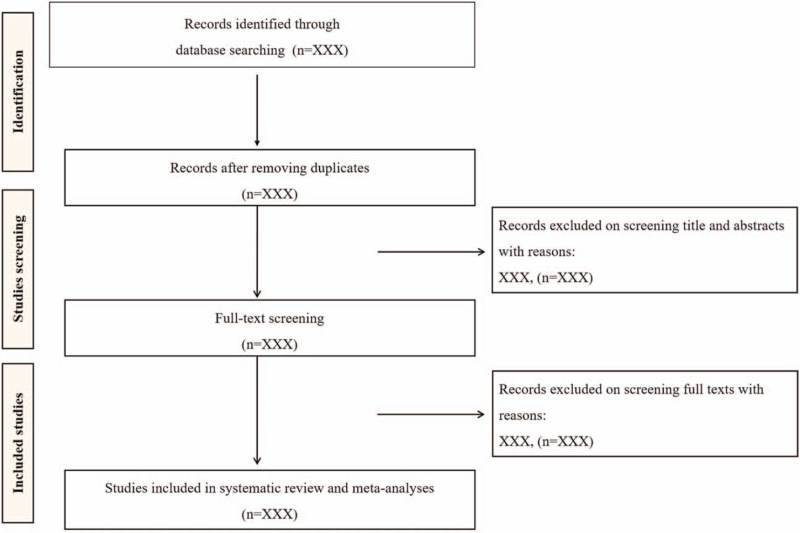
Study selection process.

Two independent reviewers will screen the retrieved articles by assessing the titles and abstracts based on the above eligible criteria. Then, the potentially eligible articles will be further assessed by the full text. The process of the study selection will be presented in Fig. 1. Disagreements will be resolved through discussion between reviewers.

### 2.5 Data extraction

Two reviewers independently extract data from the included articles. The following data will be extracted: reference information (first author, year of publication, etc), study characteristics (objectives of the study, method of randomization, method of blinding, etc), participant characteristics (ample size, age, sex, characteristics of KOA, etc), interventions (type of traditional Chinese non-pharmacological therapy, control interventions, time and frequency of intervention, follow-up time, etc), outcomes measure, and adverse effects. Any discrepancies will be resolved by discussion between reviewers.

### 2.6 Risk of bias assessment

The quality of eligible studies will be evaluated by 2 independent reviewers using the Cochrane Risk of Bias Tool for RCT: Selection bias, performance bias, attribution bias, reporting bias, and other bias, which will be divided in to “low risk of bias,” “high risk of bias,” or “unclear.” Any disagreement will be resolved through discussion.

### 2.7 Data analysis

The meta-analysis will be conducted with a random or fixed effect model to calculate the standardized mean difference and 95% confidence intervals based on different heterogeneity using the Review Manager Version 5.3 software (The Nordic Cochrane Centre, Copenhagen, Denmark). The test of *I*^2^ will be used to identify the heterogeneity. When *I*^2^-values >50%, *P*-value <.05, there will be heterogeneity among the studies. The random effect models will be used and the source of heterogeneity should be analyzed. The corresponding authors will be contacted to get detailed information if relevant data are not reported.

#### 2.7.1 Subgroup analysis

The subgroup analysis will be carried based on different types of traditional Chinese non-pharmacological therapy and different outcomes. The patients with KOA will be divided into young (<40 years) and old (≥40 years) KOA subgroups according to the age.

#### 2.7.2 Sensitivity analysis

Sensitivity analysis will be used to assess the reliability of the combined results of meta-analysis for each outcome index.

#### 2.7.3 Grading the quality of evidence

The overall quality of evidence for each outcome will be evaluated using the Grades of Recommendation, Assessment, Development and Evaluation including the risk of bias, inconsistency, indirectness, imprecision, and publications bias, which will be classified into very low, low, moderate, or high judgment.

## 3 Discussion

KOA is the most common degenerative osteoarthropathy characterized by joint pain, deformity, and limitation of activity, which seriously affects the daily life quality of patients.^[1]^ With the increasing aging of the global population, KOA will seriously threaten patients to work and live healthily, and causes a huge economic burden to patients and society. Therefore, appropriate treatments are very important for KOA sufferers especially in reducing chronic pain and functional limitation. The current systematic review will conduct a comprehensive literature and meta-analysis to evaluate the effects on pain and disability of traditional Chinese non-pharmacological therapy in the management of KOA. The primary outcomes will be focused on pain and disability of patients with KOA. The second outcomes include quality of life and adverse events. The quality of eligible studies will be assessed by the Cochrane Risk of Bias Tool for RCT. The overall quality of evidence for each outcome will be evaluated using the Grades of Recommendation, Assessment, Development and Evaluation. We believe that the current systematic reviewer and meta-analysis will attract the attention of patients with KOA and physiotherapist.

There are, however, some potential limitations in the current systematic review and meta-analysis. First, there are different traditional Chinese non-pharmacological therapies including acupuncture, moxibustion, Tuina, Tai Chi, etc. The subgroup analysis will be conducted based on different non-pharmacological therapies, but there is still potential heterogeneity. In addition, the eligible studies will be limited to English and Chinese due to language ability of the reviewers.

## Author contributions

**Conceptualization:** Wei Chang, Weina Guo, Rongjun Wang, Sifei Sun.

**Funding acquisition:** Wei Chang, Rongjun Wang.

**Methodology:** Wei Chang, Weina Guo, Xingxi Lin.

**Project administration:** Rongjun Wang, Sifei Sun, Yibin Shi.

**Writing – original draft:** Wei Chang, Weina Guo, Rongjun Wang.

**Writing – review & editing:** Wei Chang, Sifei Sun.
